# Simulation of Genes and Genomes Forward in Time

**DOI:** 10.2174/138920210790218007

**Published:** 2010-03

**Authors:** Antonio Carvajal-Rodríguez

**Affiliations:** Departamento de Bioquímica, Genética e Inmunología, Universidad de Vigo, 36310 Vigo, Spain

## Abstract

The importance of simulation software in current and future evolutionary and genomic studies is just confirmed by the recent publication of several new simulation tools. The forward-in-time simulation strategy has, therefore, re-emerged as a complement of coalescent simulation. Additionally, more efficient coalescent algorithms, the same as new ideas about the combined use of backward and forward strategies have recently appeared. In the present work, a previous review is updated to include some new forward simulation tools. When simulating at the genome-scale the conflict between efficiency (i.e. execution speed and memory usage) and flexibility (i.e. complex modeling capabilities) emerges. This is the pivot around which simulation of evolutionary processes should improve. In addition, some effort should be made to consider the process of developing simulation tools from the point of view of the software engineering theory. Finally, some new ideas and technologies as general purpose graphic processing units are commented.

## INTRODUCTION

The importance of in silico approaches in systems biology and evolutionary studies is translated in an increase of both development of new simulation algorithms and in an increasing number of reviewing works [1-4] trying to deal with the fast growing field of genetic populations simulation. Therefore, the key role of simulation software in current and future evolutionary and genomic studies has been recently emphasized, and its use in research is becoming a common place. Moreover, the expectation on increasing use of forward simulation and the improvement of algorithms [4] seems to be plenty fulfilled as demonstrated by the several new forward simulation tools just recently published in less than a year [5-10]. In addition, more efficient coalescent algorithms continue to appear [11], the same as new ideas about combined use of backward and forward strategies [8]. There are also model-based and data-guided simulation tools oriented to test the performance of disease-marker association studies, genome annotation, assembly and alignment tools [3, 12]. Given this plethora of simulation possibilities, the importance of simulation is not under discussion and forward strategy has already emerged as an alternative to coalescent simulation [3, 4, 8, 13]. 

In the present work, an update of a previous review [4] is performed. This is necessary because that review has become rapidly outdated due to the above exposed causes. Therefore, new forward simulation tools including some new algorithms will be analyzed. The conflict between efficiency and flexibility, i.e. execution speed and memory usage, versus the capability to model complex demographic scenarios will be also considered. Possibly, this will be the pivot around which simulation of evolutionary processes should improve. It is proposed as well that some effort should be made to approach the development of genetic simulation programs from the point of view of standard software engineering techniques [14]. This should help to develop more useful tools for the research community, facilitating a systematic and professional development, operation, and maintenance of such tools.

## GENETIC SIMULATION OF POPULATIONS

Simulation of genetic data under plausible evolutionary scenarios is useful to gain insight about the effect of evolutionary and demographic parameters over the sampled genetic data and also to test genetic analysis methods [3, 4]. In recent years, the testing of genetic analysis methods has been extended to include disease-marker association studies, genome annotation, alignment and assembly tools [3, 12]. Currently, sequence simulation is an important method to test any of such new genome tools. This occurs because events as compositional bias, phylogenetic correlations, heterogeneous substitutions, indel rates, context-dependent mutation etc, change the information attached to the DNA sequence reducing the effectiveness of annotation and assembly tools [12]. Some of these methods have been based on simulating the phylogenies under more or less complex models of DNA sequence evolution, for example DAWG [15] allows for the inclusion of indels the same as SIMGENOME [12], which also includes integrated parameter estimation. EvolSimulator [16] is also phylogenetic based model, which evolves prokaryote genomes allowing lateral gene transfer. The above methods are specially focused on the impact of the different mentioned evolutionary facts onto the alignment methods at the genome scale. The common feature about these models is that they evolve sequences through a phylogenetic tree given an evolutionary Markovian or hidden Markovian model. However, such programs do not consider the evolution at population level with a mating system, mutation, recombination etc. An exception seems to be EvolSimulator, which study the effects of some evolutionary events onto the phylogeny jointly with settings at the population level. On the other side, the coalescent has been the most extended model-based method of population genetics simulation in the last decade, however, the exact coalescence method loss its efficiency when simulating genomes (megabase scale) with recombination [11]. This has led to new coalescence approaches allowing a more efficient simulation of large genome regions with recombination. GENOME, for example, simulates the genealogy in a generation-step basis instead of time-to-next-event basis. SMC (Sequentially Markov Coalescent) [17] and MaCS (Markovian Coalescent Simulator) [11] simulate the genealogy beginning with a local tree that is constant for a given region flanked by two successive recombination events. Recombination is always considered as a Markovian process i.e. the modification of the given tree is independent at each recombination time in the case of SMC and only with recombination events that are far apart in the case of MaCS. Other modifications of previous coalescent programs continue to appear to include more complex models or situations, as for example selection at biallelic loci [18]. In any case, if the interest is focused on simulating evolution under complex and realistic evolutionary and demographic scenarios, the, less efficient, forward simulation should be preferred [4]. Therefore, a conflict exists between efficiency and flexibility, the more complex the model the less efficient the simulation, and vice versa. The aim should be to get complex simulation models keeping efficiency as much as possible. 

## A BRIEF VIEW ON FORWARD POPULATION GENETIC SIMULATORS

In Table **1** some forward simulators are given jointly with their web links and the programming language, in which they are implemented. Since many of them have already been reviewed [3, 4] and they are all available *via *web, here I will just briefly emphasize some of them. BottleSim simulates population bottlenecks, it includes an overlapping generation model [19]. EasyPop [20] allows generating genetic data for haploid, diploid, and haplodiploid organisms under a variety of mating systems. EvolveSimulator [16] simulates prokaryote genomes without recombination focusing specially in lateral gene transfer. FPG [21] simulates a broad range of conditions including natural selection, recombination, and migration, however, is somewhat limited by the genome size it can manage. FREGENE [5] simulates complex demographic and evolutionary models similarly to GenomePop [9]. KernelPop [22] and NEMO [23] implement individual-based, spatially explicit models. KernelPop uses the R environment [24]. Mendel's Accountant [25] performs forward-time population simulations and can be supported on parallel cluster computers. quantiNEMO [26] allows to investigate the effects of selection, mutation, recombination, and drift on quantitative traits. SFS_CODE [7] allows for a context dependent mutation model (including CpG-effects), synonymous and non-synonymous mutations, etc. SimuPop [27] simulates complex demographic and evolutionary models. Including R/Splus-like environment the users can program their own scripts in Phython. It can be supported on parallel cluster computers. The great advantage of SimuPop is its flexibility, which permits fast evolution of the program to include new features as for example non-random mating models [28] and, hence, permits the interested users to perform their own models. The main disadvantage of SimuPop is due to the aforementioned trade-off between flexibility and efficiency. The scripting or dynamic programming language has the inconvenience of less efficiency than other non dynamic languages as C++ and this will be evident when largest population sizes, sequence length and/or generation number must be simulated. On the other side, it is forwsim [8], which implements a very efficient simulator but with somewhat limited model options. To do so, it uses a forward-backward scheme i.e. exploits the genealogical information of several generations instead on a one-generation basis and building on such information simulates only the chromosomes that will contribute to the future population. Therefore, forwsim could imply a gain in time efficiency of up to one order of magnitude when comparing with other forward simulation tools [8]. However, such gain is done at the cost of only one recombination event per meiosis and limited options for mutation, migration, and selection models. Other programs as Fregene and Genomepop present a good compromise between efficiency and flexibility. Fregene [5] is oriented to genetic epidemiology allowing for ascertained gene sampling *via *the accompanying program Sample. It performs various forms of selection permitting to track the history of sites under selection. As a drawback, the program only manages diallelic SNPs data. Genomepop does not perform ascertained gene sampling, although can manage both diallelic and more complex nucleotide or codon models. However, the later is done at the cost of slightly less efficiency.

## A MODEL FOR THE SIMULATOR REQUIREMENTS DOCUMENT (SRD)

This section borrows some concepts from software engineering theory [14] and modify them to apply to the specific case of development of free and public simulation tools for the field of evolutionary biology. It is true that a new software tool is typically published as a computer note in some scientific journal. However, this note is usually just a brief description of the tool with, at best, some methodological explanation and an example case. The goal from the software engineering view should be to specify as much as possible the needs or conditions to meet for the new simulator. This will help both the developer and the researcher. That is, the specification should explain exactly what the software performs so that the user can take advantage on that. In addition, and because the interest is most of the times not commercial but scientific, it will also be desirable to explain how the software executes the task in order that anyone, if desired, can reproduce and/or improve it by himself. There are some standards for such kind of documents as that of European Space Agency [30, 31]. Of course a given evolutionary biology simulation project should be very much simpler than a given system for industry but we can borrow some useful ideas from that kind of documents.

I am proposing the simulation requirements document (SRD) that should be a complete description of the behavior of the system to be developed (Table **2**). It should include a set of use cases, also called functional requirements, describing as much as possible the interactions that the users will have with the software. In addition, the SRD also contains nonfunctional (or supplementary) requirements as performance, accessibility, availability, limit values etc. After the development of the program, the SRD should help the users to know what the software exactly can and cannot do. In Table **2**, a list of possible items included in the document is given. We distinguish five main parts in the SRD: Introduction, General Description, Specific Requirements, Design, and Verification & Validation.

### Introduction 

This section just gives a brief overview on the whole SRD. It can be subdivided in Objective, i.e. the global goal of the project and for who is intended the program; Field or context, i.e. brief description of what the program can and cannot do and the context where the software applies possible benefits of doing so. Finally, a reference list and an Index of the remaining sections of the SRD should be given. Optionally a glossary of terms and abbreviations can also be attached to this introductory section.

### General Description

This section should give a general view onto the system, including the context where the project apply, the version and relationship with previous projects, objectives and main tasks that the software is able to perform. Putative links or pipelines with other software can also be mentioned here the same as general restrictions i.e. software platform, hardware, programming language etc. A description of the conceptual model that the system develops including main sub-models, data structures, and input-output formats can also be given here. An example of the later could be: “forward-in-time, spatially-explicit software with three main sub-modules, input, evolution, output. The input format will be fasta-like the same as the output and the genetic information will be biallelic as the software manages bit strains to represent the different loci”.

### Specific Requirements

Detailed list of what the system is supposed to do. It can be divided in functional and technical requirements. Functional will include input, output, and important functions that the software should perform. For example: “the program should read sequence data in fasta-like format with each sequence identifier beginning with > and the sequence being a set of 0 and 1’s. The sequence identifier should appear in different line that the sequence, etc. The output is in the same format as the input. The program simulates a forward-in-time evolutionary model at the megabase scale”. Here, different important functions can be explicitly mentioned as mutation, recombination, migration, etc. Different sets of use cases should be given describing the potential interactions of the user with the software. Technical requirements refer to the program minimum and maximum capacities e.g. “the program will be able to run a population of minimum *N *= 2 to a maximum of *N* = 10^6^ individuals with a 100 megabase genome during 6*N* generations in less than an hour in a personal computer with 1 Gb ram. Higher values of *N* will run more or less efficiently depending on the processor and memory requirements. Recombination values as low as 0 and as high as 0.5 can be used…” etc. More technical information, as type of memory allocation needed to fulfill specific data management etc, should also be given here, though how the specific implementation of such structures is done corresponds better with the next section (design).

### Design

This section could be included in the SRD or could be an independent document by itself. The section should provide a detailed definition and structure of the system so that any developer other than the designer can reproduce it. For example, if an object oriented design is used, the structure of classes should be given here. More specific information on data structures will also be given here if it was not done in the model description section above. Finally, the source code can accompany this section. Giving the source code, however, should not substitute the specific and detailed information in the Design section. 

### Verification & Validation

This section of the SRD will explain how the software was tested and validated. Verification tries to ensure that the software works right, for example check that the processes of mutation and recombination are working as expected. Validation ensures that the software performs what was intended for. The last could seem quite obvious in this context but having explicitly this item could help on the success of the project. Consider for example a case where the specified goal was to evolve genomes under complex models of evolution and demography. To validate such goal it is not enough that the program runs in assumable time a few sequence kilobases but at least should do that at the megabase scale. 

## DISCUSSION

Due to the high number of recent simulators, especially forward ones, the potential user can be unsure which of them to choose. Additionally, it could be that no simulator adapt to the specific necessities. Unfortunately, it is not uncommon that the user has difficulties on knowing exactly what the software can and cannot do. 

As simulation tools become more flexible, the incorporation of some standards for documentation seems to be important because it will facilitate both the development of more useful tools for the research community jointly with a systematic and qualified improvement, operation, and maintenance of such tools. For example, performing the SRD for a given simulation project should try to answer questions like, kind of marker to model, implications of modeling such one, what evolutionary and demographic contexts are noteworthy given it. How the input and output should be, and what programs, if any, would be interesting to pipeline with the new tools. Specifying the functional requirements part will help to think about most efficient ways of performing mutation, recombination, and migration, which will be reflected in the design section, but will also help the user to know exactly what specific models he/she can and cannot run using such program. Obviously, the target is always to get software as good as possible regarding properties as maintainability, reliability, efficiency, and usability [14].

With the advent of more powerful computers and the memory cheapening, the trade-off between flexibility and efficiency is continuously being reset by more powerful and flexible programs, which also improve efficiency with respect to previous ones. However, as far as more and more genomic and proteomic information is at hand the conflict prevails, consider for example the kind of new algorithms combining backward and forward simulation gaining the necessary efficiency for managing genomes in assumable time but somewhat loosing the characteristic flexibility of forward-in-time simulation. Hence, new technologies could contribute to the improvement of algorithms to allow both high flexibility and efficiency. One of such is the modern graphical process units (GPUs), which are specialized processors with highly parallelized structure that makes them very efficient for floating point calculations. One of this parallel computing architecture is Cuda, which interestingly provides the possibility to code algorithms for execution on the GPU *via *standard programming languages as C by using some language extensions as the Cuda programming environment recently released by NVIDIA [32]. Such programming environments that allow the use of GPUs for general purpose programming, the so-called GPGPUs [33] are beginning to be employed for solving computational biology oriented problems [34, 35] with improvements of up to two orders of magnitude with respect to standard CPU implementations.

## Figures and Tables

**Table 1.** Forward Simulators

Name	Web link	PL
BottleSim	http://chkuo.name/software/BottleSim.html	C++
EasyPop	http://www.unil.ch/dee/page36926_fr.html	C
EvolSimulator	http://www.bioinformatics.org.au/evolsim/	C++
ForSim [6]	http://www.anthro.psu.edu/weiss_lab/research.shtml#ForSim	C++
forwsim	http://www.people.cornell.edu/pages/bp85/	C++
FPG	http://lifesci.rutgers.edu/~heylab/HeylabSoftware.htm#FPG	C
FREGENE	http://www.ebi.ac.uk/projects/BARGEN/download/FREGEN/documentation_html.html	C++
GenomePop	http://webs.uvigo.es/acraaj/GenomePop.htm	C++
KernelPop	http://cran.r-project.org/web/packages/kernelPop/index.html	C++R
Mendel's Accountant	http://mendelsaccountant.info/	F, C
NEMO	http://sourceforge.net/projects/nemo2	C++Java
quantiNEMO	http://www2.unil.ch/popgen/softwares/quantinemo/	C++
Rmetasim [29]	http://linum.cofc.edu/software.html	C++ R
SFS_CODE	http://cbsuapps.tc.cornell.edu/sfscode.aspx	C
SimuPop	http://simupop.sourceforge.net/	P C++

PL: Programming Language. F: Fortran. P: Phyton.

**Table 2.** The Simulator Requirements Document (See Text for Details)

Simulator Requirements Document
**Introduction**
Objective
Context
Index
**General Description**
Context
Main objectives and tasks
Connection with other software
Model
Formats
**Specific Requirements**
Functional
Technical
**Design**
Classes, Objects
**Verification & Validation**
Test cases, Fulfilled goals
